# Patients' and nurses' experiences of fundamental nursing care: A systematic review and qualitative synthesis

**DOI:** 10.1111/jocn.15082

**Published:** 2019-11-19

**Authors:** Claire Pentecost, Julia Frost, Holly V. R. Sugg, Angelique Hilli, Victoria A. Goodwin, David A. Richards

**Affiliations:** ^1^ Institute of Health Research University of Exeter Medical School Exeter UK

**Keywords:** elimination, experience of care, fundamental care; qualitative synthesis, hygiene, mobility, nurses, nutrition, patients

## Abstract

**Aims and objectives:**

To systematically identify, appraise and synthesise patients**'**, residents**'** and nurses**'** experiences of fundamental nursing care for nutrition, elimination, mobility and hygiene.

**Background:**

The evidence base for effective nursing behaviours to assist people with their fundamental care needs is sparse, hampering the development of effective interventions. Synthesising data on patients**'** and nurses**'** experiences of fundamentals of nursing care could contribute to the development of such an intervention.

**Methods:**

Systematic review and synthesis of qualitative data from qualitative studies on patients**'** and nurses**'** experiences of fundamental nursing care behaviours addressing peoples**'** nutrition, elimination, mobility and hygiene needs. We appraised study quality and relevance and used a narrative approach to data synthesis, fulfilling PRISMA criteria (Appendix S2).

**Results:**

We identified 22,374 papers, and 47 met our inclusion criteria. Most papers were of low quality. Sixteen papers met our quality and relevance criteria and were included for synthesis. Papers were about nutrition (2) elimination (2), mobility (5), hygiene (5) and multiple care areas (2). We found nurses and patients report that fundamental nursing care practices involve strong leadership, collaborative partnerships with patients and cohesive organisational practices aligned to nursing care objectives and actions.

**Conclusions:**

To improve fundamental care and interventions suitable for testing may require attention to leadership, patient–nurse relationships and organisational coherence plus the fundamentals of care nursing interventions themselves.

**Relevance to clinical practice:**

More rigorous mixed methods research about fundamental nursing care is needed to inform nursing practice and improve patient**'**s experience. Nursing interventions should include effective nurse leadership and nurse–patient collaboration and a focus on fundamental care by the host organisation.

**What does this paper contribute to the wider global clinical community?**

- We have identified preferred nursing practices in four essential care areas, nutrition, elimination, mobility and hygiene.
- High‐quality and relevant studies have been synthesised, and three conceptual themes were identified: nurse leadership, partnerships with patients and organisational practices.
- Nurse leadership and organisational practices need to demonstrate prioritisation of partnerships with patients in delivering essential nursing care in order that nursing care quality and patients experience of care is improved.
- We will use the framework from our Amalgamation of Marginal Gains logic model to incorporate this knowledge and design our fundamentals of care nursing intervention to be empirically evaluated.

## 1 INTRODUCTION

### 1.1 Background

Nursing care is an essential element of healthcare provision and has a direct and significant impact on patient outcomes (Rathert, Wyrwich, & Boren, 2013). Unfortunately, when nursing care is done poorly or is missing there are serious consequences (Aiken et al., 2014; Ball et al., 2016; Department of Health, 2012b, 2013). Improving patient experience of care through “person‐centred care” is widely promoted as an opportunity to improve quality of care and patient outcomes (Ahmad, Ellins, Krelle, & Lawrie, 2014; Ball, Murrells, Rafferty, Morrow, & Griffiths, 2014; de Silva, 2014) by placing patients' experiences at the heart of care.

Consequently, a number of significant initiatives have attempted to refocus nursing care on the essential principles of nursing practice (Department of Health, 2012a). This attention to “fundamental nursing care” has gained international attention from the nursing profession (Blomberg, Griffith, Wengstrom, May, & Bridges, 2016; Feo & Kitson, 2016; Kitson, Conroy, Kuluski, Locock, & Lyons, 2013). Fundamentals of care are defined as follows: action to address safety, comfort, communication, dignity, respiration, privacy, eating and drinking, respecting choice, elimination (toileting), mobility, personal cleansing and dressing, expressing sexuality, temperature control, rest and sleep (Kitson, Conroy, Wengstrom, Profetto‐McGrath, & Robertson‐Malt, 2010). These fundamentals are seen as the essence of nursing care.

Despite a heightened awareness of the importance of fundamental nursing care, the existing nursing literature has been criticised for an absence of research evidence to guide practising nurses (Chalmers & Glasziou, 2009; Hallberg, 2009; Kitson, Muntlin Athlin, & Conroy, 2014; Richards, Coulthard, & Borglin, 2014). Coupled with a lack of empirically tested theoretical models of care (Dewing & McCormack, 2017), there is an almost complete lack of evidence for effective nursing care in any of the foremost key fundamental areas of nutrition, hygiene, mobility or elimination (Richards, Hilli, Pentecost, Goodwin, & Frost, 2018). There is a clear need to develop both the constituent scientific basis and consequent clear evidence‐based guidelines that can be used by the profession in the delivery of fundamental patient care.

This paper is a component part of the ESSENCE (amalgamation of marginal gains in Essential Nursing Care) programme of research aiming to develop a complex fundamental nursing care intervention (Craig et al., 2008; Moore et al., 2015). Our nursing intervention is based on a model for improving performance used in sport and health care called Amalgamation of Marginal Gains (AMG) (Richards, 2015), a process of finding many candidate small improvements and making changes that when combined have a large impact on the desired outcome (Richards, 2015). In our previous work to understand how AMG has been applied to improve performance, we determined that AMG included whole group or team desire to achieve an overarching objective, a process of identification and selection of marginal gains, implementation of marginal gains changes with monitoring, feedback and regular review, and leadership to drive new practices (Pentecost, Richards, & Frost, 2018).

Our innovative nursing intervention will incorporate synthesised evidence from our systematic review of effective candidate fundamental nursing behaviours (Richards et al., 2018), a logic model derived from our qualitative data on the key processes of successful AMG innovation (Pentecost et al., 2018) and, finally, the results of a synthesis of qualitative studies identified in our systematic review (Richards et al., 2018) presented here in this third paper. In this final study, we aimed to elicit data on factors that impact on the quality and experiences of care in the essential areas of hygiene, mobility, elimination and nutrition, and potential mechanisms of interventions to construct an explanatory model of relationships between the core concepts identified (Frost, Garside, Cooper, & Britten, 2016). These three studies will, therefore, provide the evidence to underpin the development of our intervention (Moore et al., 2015).

## 2 AIMS AMD METHODS

### 2.1 Objective

To systematically identify, appraise and synthesise qualitative data from primary empirical studies about patients', residents' and nurses' experiences of nursing care of nutrition, elimination mobility and hygiene needs in order to identify any overarching conceptual themes, and to construct an explanatory model of relationships between concepts that must be considered in our intervention design.

### 2.2 Review question

What are the overarching thematic concepts that can be synthesised from the views of patients, residents and nurses captured in primary qualitative studies on their experience of receiving and delivering fundamental care in the areas of nutrition, elimination mobility and hygiene in the qualitative literature?

### 2.3 Design

We undertook a systematic review and synthesis of primary qualitative studies (Popay et al., 2005) by (a) identifying studies, appraising the quality and relevance of study data and synthesising that data following established methods for reviewing qualitative literature (Popay et al., 2005; Pope, Mays, & Popay, 2007) and (b) establishing relevance of data to our study objective (Britten, Garside, Pope, Frost, & Cooper, 2017). We followed PRISMA checklist criteria (Moher, Liberati, Tetzlaff, Altman, & The PRISMA Group, 2009) (Appendix S2) when conducting and reporting this study (See Appendix S2).

### 2.4 Information sources and searching

We searched relevant databases to ensure as comprehensive as possible a body of literature to synthesise (Popay et al., 2005) during a period of time from May 2015–March 2016. We searched EMBASE, MEDLINE, CINAHL, PsychLIT, PsycINFO, CANCERLIT, Science Citation, the COCHRANE library, using the OVID MEDLINE^®^ platform, and individual database searches. We used broad search criteria to allow us to identify papers that met the criteria for our systematic review (Richards et al., 2018) and this qualitative synthesis. We contacted the authors of studies where we were unable to access the full‐text paper or report through online databases and journals. We hand searched the reference lists of included reviews for relevant primary papers and identified additional citations through our networks and conference attendance. We conducted individual searches for each of the essential care areas: nutrition, elimination, mobility and hygiene (Kitson et al., 2010). We used MeSH and free‐text terms adapted to each of the specific databases searched. An example of one of the search strategies is presented in Appendix S1. Other searches are available from the authors upon request.

**Figure 1:**
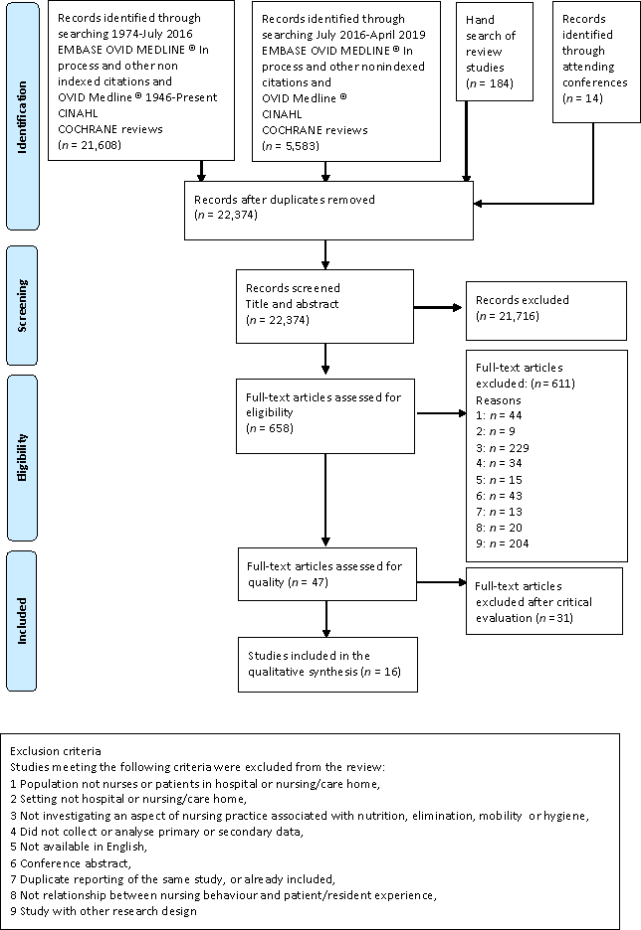
PRISMA diagram

To ensure this qualitative review includes the most recent literature, we replicated the search specifically for qualitative papers again in May 2019 using the same search terms and databases to identify any papers published between April 2016–May 2019 that met the inclusion criteria (Figure 1).

### 2.5 Data management

We uploaded the identified references for each search to EndNote™ reference management software (http://www.endnote.com) and removed duplicates. Records of the screening process were kept by retaining the EndNote™ databases for each independent reviewer at each step of the screening process.

### 2.6 Eligibility criteria

We included all qualitative research designs including those guided by an explicit set of philosophical and theoretical assumptions, those using specific qualitative methodologies and studies not underpinned by theory or that used undefined generic forms of qualitative research. We included papers written in English reporting the results of primary qualitative research studies, with data collected from patients in hospitals and residents in care homes or from registered or unregistered nurses reporting their experiences of nursing care interventions or behaviours in nutrition, elimination, mobility and hygiene. We defined studies about nursing behaviours relevant to nutrition as those to assist or support patients or residents in consuming adequate food and fluids to achieve optimum nutritional and hydration status. Care of elimination needs was defined as nursing behaviours undertaken to address the toileting needs of patients or residents. Mobility care was defined as nursing behaviours to assist or support patients or residents to move, and hygiene care was defined as care behaviours to assist or support patients or residents to maintain bodily cleanliness, hygiene and dressing.

### 2.7 Study selection

Two members of our research team (VG, CP, AH, HS) independently screened titles and abstracts retrieved in both searches to arrive at an initial set of potential studies for inclusion. We then assessed these full texts against our eligibility criteria (Popay et al., 2005). Disagreements were resolved at each stage by discussion between researchers.

### 2.8 Data extraction

Data extraction was guided by the overall aim of our research programme and our research question (Popay et al., 2005). We extracted data using an adapted version of a data extraction sheet used previously by the research team (Richards et al., 2014). Two researchers (AH, CP) for the first search and two researchers for the second (CP, HS) extracted data on lead author, year and place of publication, study origin country, essential care area, qualitative methodological orientation, setting, population studied, interventions or usual nursing care behaviours, delivery personnel, quality criteria and author findings about nurse or patient experience of nursing care. Disagreements were resolved through discussions within the research team, with any necessary dispute resolution provided by a third reviewer (DR or JF).

### 2.9 Appraisal

The critical appraisal of qualitative research is controversial (Barbour, 2001), and we therefore used several approaches to identify the most robust evidence to contribute to the development of an intervention. We appraised studies individually and reached consensus by discussion. We appraised discrete element of the papers (CP, AH, JF), such as study design, sampling and analytical techniques, to provide a global map of the quality of the literature (Croucher, Quilgars, Wallace, Baldwin, & Mather, 2003; Wallace, Croucher, Quilgars, & Baldwin, 2004). This enabled us to identify papers as having a high, low or unclear level of quality (Popay et al., 2005). The higher quality papers were then appraised (JF, AH) using the GRADE‐CERQual tool (Lewin et al., 2015) which enabled us to identify a further subgroup of key or “conceptually rich” papers within the high‐quality papers, which were those that could potentially make the most important contribution to our synthesis (Britten et al., 2017). These were identified by assessing papers for evidence of useful and effective nursing care behaviours addressing fundamental care needs from the perspective of patients and nurses that could inform nursing practice.

### 2.10 Data synthesis

The key conceptually rich papers then formed our preliminary analytical framework, to which the data in the remaining high‐quality papers were added. We employed an established narrative qualitative synthesis approach, namely developing a preliminary synthesis of the findings of included studies, developing a theory of how and why the nursing interventions did or did not work, exploring relationships in the data and assessing the robustness of the synthesis (Popay et al., 2005). We moved iteratively between these elements as our synthesis progressed.

We first synthesised definitions or examples of nursing care behaviours adopted by qualified and unqualified nurses and care staff from the perspectives of patients, nurses or researchers in each of our four care areas of interest (nutrition, elimination, mobility and hygiene needs) and examined the themes identified by the authors of the primary studies, both to familiarise ourselves with their content and to explore their scope (Popay et al., 2005). Within each of the four groups, we then used the “conceptually rich” (Malpass et al., 2009) “index papers” (Campbell et al., 2003) to develop our preliminary understanding of the nature of the themes identified by authors.

Having noted and described the key findings for each of the four domains of interest in the six conceptually rich papers, our analysis developed by “exploring relationships in the data” (Popay et al., 2005) across the wider set of the 14 high‐quality papers, for example identifying any explanations for any differences in “barriers and facilitators” to high‐quality care across the essential care areas and evidence of how authors evaluated behaviours as successful or unsuccessful (Popay et al., 2005). We identified similarities and differences between groups of studies by comparing data in the conceptually rich papers and augmented these findings with data from the remaining eight high‐quality papers, thus enriching and strengthening our conceptual understanding. This allowed us to define the *substantive* themes identified by the authors of the primary research and subsequently enabled us to identify overarching *conceptual* themes which operate across the four areas of interest by synthesising themes pertaining to factors influencing successful implementation of interventions, and care behaviours common to the four care areas and across all care areas.

We then explored and sought to define these concepts as candidate components of a future intervention. At this stage, we refocused specifically on those primary studies which were most relevant and illuminating (Lewin et al., 2015) to ensure the validity of our synthesis (Popay et al., 2005). We summarised key explanatory themes and identified higher order conceptual themes that operated across the studies.

## 3 RESULTS

### 3.1 Study identification

Of the 21,806 papers derived from our search, we identified 7 as meeting the inclusion criteria for our review after screening of titles and abstracts, and assessment for eligibility of full texts (Figure 1).

### 3.2 Scope

The 47 studies (Table 1) qualitative study designs reported were: grounded theory (*n*=9), ethnography (*n*=6), phenomenology (*n*=5), narrative case study (*n*=1), and other designs including action research (*n*=1), soft systems approach (*n*=1) and interpretative description (*n*=1). Other studies did not specify the design but described their analysis as content analysis (*n* = 8), framework analysis (*n* = 3), thematic analysis (*n* = 4) or did not specify (*n* = 8).

**Table 1:** Characteristics of qualitative studies

No	Essential care area^a^	Name, Year, Country	Methodological orientation/Theory^b^	Setting	Population: patients condition^c^ (number), method of data collection; staff^d^ (number) method of data collection	Area studied/introduction of changes such as training, or new protocol yes (y) or no (n)	Delivery personnel^e^
*Mealtime assistance*							
1	Nutrition	Dickinson (2008) UK	Other‐Action research/emancipatory framework	Hospital	Unspecified (*n* = 6), interviews; nursing and other staff (*n* = 19), interviews and observation	Mealtime assistance including prioritisation of and engagement in mealtimes and ensuring availability of sufficient time and expertise/y	MTD including Nurse(s)
2	Nutrition	Gibbs‐Ward (2005) USA	Grounded theory/none stated	Nursing home (*n* = 2)	Neurological (*n* = 20), observation; health care aides (*n* = 18) interviewed	Mealtime assistance that promotes independence/n	Both
3	Nutrition	Heaven (2013) UK	Grounded theory/none stated	Hospital (*n* = 4)	Unspecified (*n* = 5), interviewed; General staff (*n* = 54), stakeholders (*n* = 6), former patients and carers (*n* = 5), focus groups	Food work and feeding assistance including order of service, choice, and facilitation/n	MTD including Nurse(s)
4	Nutrition	Mentes (2006) USA	Content analysis/none stated	Nursing home (*n* = 3)	None; care staff (*n* = 28), focus groups	Strategies for improving hydration/n	Both
5	Nutrition	Palacios‐Cena (2013) Spain	Phenomenology/none stated	Nursing home (*n* = 4)	Unspecified (*n* = 26), interviews; none	The significance of meals for residents/n	MTD including Nurse(s)
6	Nutrition	Roberts (2014) UK	None stated	Hospital	None; Volunteer care assistants (*n* = 29),focus groups	Feeding, encouraging and assisting to eat, preparing tables, cleaning patients' hands and talking with them/y	Nurse‐unreg.
7**	Nutrition	Robison (2015) UK	Framework/none stated	Hospital	Unspecified (*n* = 15; *n* = 20) interviewed; Nursing staff (*n* = 9; *n* = 11), focus groups	Encouragement to eat, support with opening packets and setting up the meal tray, cutting up food, helping guide the food to the patient's mouth and feeding patients/y	Both
8	Nutrition	Schell (1999) USA	Grounded theory/Symbolic interaction theory	Nursing home	Unspecified (*n* = 10), observed; care staff (*n* = 32), observed	Mealtime care including addressing the patient, sharing the experience by eating with them and talking/n	Nurse‐unreg.
9	Nutrition	Sidenvall (1994) Sweden	Ethnography/none stated	Nursing home (*n* = 2)	Unspecified (*n* = 18); nurses (*n* = 21) observed and interviewed	Mealtime care including offering aids such as feeding cups/n	Both
10	Nutrition	Steele (2007) Canada	None stated	Nursing home	None; volunteer care assistants (*n* = 43) surveyed; (*n* = 7) in focus group	Mealtime care including assistance by volunteers to ensure adequate fluid intake/n	Nurse‐unreg.
11	Nutrition	Xia (2006) Australia	None stated	Hospital	Unspecified (*n* = 48); nurses (*n* = 50) observed, Unspecified staff (*n* = 4) and nurses (*n* = 4) interviewed	Observations of mealtime care/n	Both
12*	Nutrition	Sjögren Forss (2018) Sweden	Content analysis/none stated	Nursing home	Unspecified (*n* = 4), interviews; nurses (*n* = 8), interviews	Nutritional care including management of malnutrition, use of nutritional supplement drinks, involvement in mealtime environment and menu setting/n	Nurse‐reg.
*Nutritional support*							
13	Nutrition	Holst (2011) UK	Content analysis/none stated	Hospital	Unspecified (*n* = 12) interviewed; none	Patients experiences of nutritional care including motivation and guidance to maintain and increase food intake/n	MTD including Nurse(s)
14	Nutrition	Kayser‐Jones (1999) USA	Ethnography/none stated	Nursing home (*n* = 2)	Oral and Gastrointestinal (*n* = 40), observed; none	Observed factors contributing to dehydration/n	Nurse‐unreg.
						Feeding protocols	
15	Nutrition	Pasman (2003) Netherlands	None stated	Nursing home (*n* = 2)	Neurological (*n* = 60); care staff (*n* = 11) observed	Nurses reactions and actions taken to patients' adverse behaviours during feeding/n	MTD including Nurse(s)
*Multi‐component incontinence management*							
16	Elimination	Brady (2016) UK	Framework/none stated	Hospital	Stroke (*n* = 15), interviewed; nursing staff (*n* = 23), interviewed	Stroke‐specific urinary incontinence intervention including staff training, a continence assessment tool, protocol of care, improved links to continence care experts, continence assessment equipment and availability of incontinence products/y	Both
17**	Elimination	French (2016) UK	Content analysis/Normalisation process theory	National Health Service (NHS) stroke services (*n* = 8)	None; Nursing staff and other staff (*n* = 38), interviewed	A systematic voiding programme including a 3‐day bladder diary and continence assessment, bladder training to promote continence, prompted voiding to minimise incontinent episodes for those with cognitive impairment and weekly reviews of progress with change from prompted voiding to bladder training if cognitive ability improved/y	MTD inc. Nurse(s)
18**	Elimination	Thomas (2014) UK	Other‐soft systems approach/Normalisation Process Theory	Hospital	Stroke (*n* = 43); nursing staff (*n* = 18) and (*n* = 21) interviewed	A systematic voiding programme including a combination of bladder training and pelvic floor muscle training or prompted voiding, together with assessment and review of cognitive capabilities and progress/y	Unspecified
19+	Elimination	Gibson (2018) UK	Thematic analysis/ Phenomenology	Hospital	Stroke (*n* = 12) and carers (*n* = 4), interviews; none	A systematic voiding programme including a 3‐day bladder diary and continence assessment; individualised treatment plans including pelvic floor muscle training, bladder training to promote continence, individualised and prompted voiding schedule, education programme and patient‐held voiding diary/y	MTC inc. Nurse(s)
*Promoting independent mobility*							
20**	Mobility	Boltz (2011) USA	None stated/The Health Quality Outcomes Model	Hospital (*n* = 2)	None; Nurses (*n* = 43) and nonregistered nurses (*n* = 12) in focus groups	Organisational barriers and facilitators influencing physical function/n	MTD inc. Nurse(s)
21*	Mobility	Bourret (2002) USA	none stated	Nursing home (*n* = 3)	Unspecified (*n* = 23), focus groups; nursing staff (*n* = 17), focus groups	Mobility and mobility enhancing strategies and mobility limitations such as availability of appropriate assistive devices and assistance from nurses to mobilise/n	Both
22	Mobility	Doherty‐King (2013) USA	Grounded theory (dimensional analysis)/none stated	Hospital (*n* = 2)	None; Nursing staff (*n* = 25), interviewed	Views of ambulation of patients and strategies to motivate and advance patients towards independence/n	Nurse‐reg.
23	Mobility	Kindblom‐Rising (2007) Sweden	Phenomenology/none stated	Not explained	None; Nurses and nonregistered nurses (*n* = 20), interviewed	“Natural mobility” education aimed at changing communication skills in patient transfer based on theories of learning, movement awareness with and without fear, communication and basic body awareness/y	Both
*Promoting independent mobility*							
24*	Mobility	Kneafsey (2013) UK	Grounded theory/none stated	Hospital (*n* = 3)	None; Rehabilitation staff and nurses (*n* = 39) interviewed and observed	Nursing team involvement in‐patients' mobility maintenance and rehabilitation/n	MTD inc. Nurse(s)
25*	Mobility	Taylor (2014‐1) Australia	Ethnography/none stated	Nursing home (*n* = 3)	None; Unspecified (*n* = 15), interviewed, senior staff (*n* = 10) interviewed, care staff (*n* = 18), focus groups	Mobility care including transfers on and off chairs and wheelchairs, as well as assisted walking/n	MTD inc. Nurse(s)
26	Mobility	Taylor (2014‐2) Australia	Ethnography/none stated	Nursing home (*n* = 3)	None; care staff (*n* = 15) interviewed	Mobility care including transfers in and out of chairs and wheelchairs and walking/n	Unspecified
27*	Mobility	Taylor (2014‐3) Australia	Ethnography/none stated	Nursing home (*n* = 4)	None; care staff (*n* = 18), focus groups	Mobility care including transfers in and out of chairs and wheelchairs and walking/n	MTD inc. Nurse(s)
						Falls risk reduction	
28	Mobility	Barber (2015) Australia	Content analysis/none stated	Hospital	None; ICU clinicians and nurses (*n* = 25), focus groups	Early mobilisation including helping patients to sit out of beds, standing up and walking/n	MTD inc. Nurse(s)
*Cleaning people*							
29	Hygiene	Bradway (2010) USA	Content analysis/none stated	Nursing home (*n* = 2)	None; care staff (*n* = unspecified) interviewed	Continence care including bathing, washing and dressing/n	Both
30*	Hygiene	Coyer (2011) USA	Thematic analysis/none stated	Hospital (*n* = 4)	None; nurses (*n* = 42), focus groups	Hygiene care including bed baths/n	Nurses‐reg.
31*	Hygiene	Gaspard (2012) Canada	Thematic analysis/none stated	Nursing home (*n* = 12)	None; care staff (*n* = 18), focus groups	Bathing strategies seen to be successful/n	Nurses‐unreg.
32	Hygiene	Gibb (1990) Australia	Grounded theory/none stated	Nursing home (*n* = 2)	None; care staff (*n* = 10), observed	Communication during morning care including explanation, questions, confirmation/n	Nurses‐reg.
33	Hygiene	Jackson (2014) USA	Framework/none stated	Hospital	None; Nurses (*n* = 20), interviewed	Infection prevention behaviours when washing patients/n	Nurses‐reg.
34	Hygiene	Lezzoni (2012) USA	Other – Narrative case study/none stated	Hospital (*n* = 4)	Neurological (*n* = 1), interviewed; none	Hygiene care/n	Unspecified
35	Hygiene	Miller (1997) USA	None stated	Nursing home	None; care staff (*n* = 27), interviewed	Responses to patients aggressions during hygiene care/n	Both
*Oral hygiene*							
36	Hygiene	Chalmers (1996) Australia	None stated	Nursing home (*n* = 11)	None; care staff (*n* = 488) surveyed (*n* = 65), interviewed	Denture and teeth cleaning, mouth rinse, flossing and swabbing/n	Nurses‐unreg.
37	Hygiene	De Visschere (2015) Belgium	Thematic analysis/none stated	Nursing home (*n* = 13)	None; care staff (*n* = 36), interviewed	Cleaning teeth and dentures/n	Both
38	Hygiene	Sonde (2011) Sweden	Content analysis/none stated	Nursing home (*n* = 9)	None; Care staff (*n* = 9) in focus groups, Nurses (*n* = 4), interviewed	Description of oral care including preferred strategies and factors influencing oral care/n	Both
39*	Hygiene	Wardh (2000) Sweden	Grounded theory/none stated	Nursing home (*n* = 3)	None; Care staff (*n* = 8) Home care aides (*n* = 14), interviewed	Description of oral care including cleaning teeth twice a day, wearing gloves during activities, visits by dental teams, taking care of dentures, explaining the content and purpose of oral care for patients/n	Nurses‐unreg.
*Assisted body‐care*							
40**	Hygiene	Jensen (2013) Denmark	Other‐Interpretive description/none stated	Hospital	Cardiovascular (*n* = 11), interviewed; none	“Patient centred personal body care” where patients take an active part, nurses adopting an attentive participating and wait and see attitude, normal time pressures are absent, informing patients of actions to be taken and increasing their awareness of both their own capability and of the nursing staff's competences to help them feel secure/y	Both
41**	Hygiene	Lomborg (2005) Denmark	Grounded theory/symbolic interactionism	Hospital	Cardiovascular (*n* = 12), observed and interviewed; none	Assisted personal body care by washing the whole body, including teeth, hair, dressing and, for men, shaving/n	Both
42	Hygiene	Lomborg (2008) Denmark	Grounded theory/Symbolic interactionism	Hospital	Cardiovascular (*n* = 12); nurses (*n* = 4), interactions observed	Assisted personal body care by washing the whole body, including teeth, hair, dressing and, for men, shaving/n	Both
*Clothing*							
43	Hygiene	Edvardsson (2009) Sweden	Phenomenology/none stated	A hospice, an in‐patient geriatric unit, an acute medical unit, and an in‐patient oncology unit	Unspecified (*n* = 9), interviewed; care staff (*n* = 5), interviewed	Meanings of wearing patient clothing including health care staff giving patients clothing to wear and changing them as needed with clean ones/n	Unspecified
*Usual care*							
44	2+FOC	Kalish (2012) USA	Phenomenology	Hospital (*n* = 2)	Unspecified (*n* = 38) interviewed; none	Nursing practices of mouth care, ambulation, getting out of bed into a chair, informing patients, bathing, listening to patients' questions and concerns, answering call lights, fulfilling requests and helping patients to bathrooms as needed/n	Unspecified
45*	2+FOC	Kitson (2013b) Australia	Phenomenology (secondary analysis)	Multiple healthcare settings	Stroke (*n* = 15) interviewed; none	Management of the fundamentals of care needs (e.g. elimination, personal hygiene, eating,) including integrating and coordinating the physical, psychosocial and relational dimensions of care around the needs of patients/n	Unspecified
46	2+FOC	Wolf (1993) Not given	Ethnography	Hospital	Patients, nursing staff, family members and other hospital personnel (*n* = 32), observed and interviewed	Nursing rituals including bathing and bed baths/n	Both
47+	2+FOC	Lafrenière (2017) Canada	Content analysis/none stated	Acute‐care University Hospital	Internal medicine (*n* = 30), interviews; none	Strategies used to prevent functional decline in older patients including addressing mobility, nutrition and hydration, urinary continence and regular bowel movements	Unspecified

^*^ Studies rated as high quality.

^**^ Studies rated as high quality, considered conceptually rich and had an important contribution to synthesis according to Grade‐CERQual assessment.

^+^ Papers added from second search.

a Essential care area: Nutrition; Elimination; Mobility; Hygiene; studies addressing care as usual focussing on two or more fundamental care areas (2+FOC).

b Methodological orientation and Theory: Grounded theory, Discourse analysis, Ethnography, Phenomenology, Content analysis, Other ‐, None stated.

c Population Typology of patients based on http://www.hrcsonline.net/hc/summary Staff: nursing staff ‐ unspecified qualifications of nursing staff, nurse‐qualified nurse, non‐registered nurse – prequalification nurse or nursing assistant, care staff –unspecified qualifications of staff responsible for patient care.

d Delivery personnel: registered nurse (Nurse‐reg.); unregistered nurse (Nurse‐unreg.); both registered and unregistered nurses (Both); multi‐disciplinary team including nurse(s) (MTD).

e Grade‐CERQual based assessment: conceptually rich papers that had an important contribution to synthesis.

Data collection included interviews (*n* = 21), focus groups (*n* = 9), observations (*n* = 6) or a combination of these (*n* = 11). Settings were hospital (*n* = 23), nursing homes or care homes (hereafter care homes) (*n* = 20), outpatient stroke services (*n* = 2) or a combination of hospital and nursing home (*n* = 1), or not described (*n* = 1). Studies collected data from nurses, nonregistered nurses or nursing home care staff (*n* = 25), patients in hospitals or residents of care homes (*n* = 9) or both patients and nurses, or residents of care homes and care staff (*n* = 12). Three studies also collected data from other groups including former patients, carers and family members. Where the clinical condition of the hospital patient was given (*n* = 8), these were people with stroke (*n* = 4), cardiovascular disease (*n* = 3) and neurological conditions (*n* = 1). In care home settings, where reported, the clinical condition of the resident participants was neurological (two studies) and gastrointestinal (one study).

Regarding the care areas studied, 15 studies were about hygiene, nine about mobility, four about elimination, 15 about nutrition and four about more than two essential care areas. Hygiene studies were categorised as cleaning people (*n* = 7), oral hygiene (*n* = 4) and assisted body care (*n* = 3). Within the area of mobility, studies were categorised as “promotion of independent mobility” (*n* = 8), and one was about “falls risk reduction.” Each of the four elimination studies was multi‐component incontinence management studies. Within the area of nutrition, studies were categorised as “mealtime assistance” (*n* = 11), “nutritional support” (*n* = 3), “feeding protocols” (*n* = 1) and “wearing clothing” (*n* = 1). Four studies observed usual care of more than one essential healthcare area. Ten of the 47 studies were experiments to manipulate nurse's behaviour by introducing new protocols and/or new training for nurses.

### 3.3 Quality

The quality of the studies was mostly low. Only 16 of the 47 papers satisfied quality criteria sufficiently to be included in our synthesis (Figure 1). The papers that were rated as high quality more often had a combination of a clear research question, clear theoretical underpinning, an appropriate study design to answer the research questions and adequately reported data collection and/or analysis and so were rated as having low risk of bias. Low‐quality studies in comparison had more missing information, more unclear information especially about methods and analysis were rated as having high risk of bias. The quality criteria and assessment for all 47 papers can be found in Table 2.

**Table 2:** Quality assessments

No	Name, Year, Country	Essential care area	Intervention	Study quality elementᵇ										Overall qualityᶜ
				Q1 E	Q2 D	Q3 E	Q4 E	Q5 D	Q6 E	Q7 E	Q8 D	Q9 D	Q10 D	
1	Dickinson (2008) UK	Nutrition	Self‐care	−	+	+	−	−	−	+	+	U	+	UNCLEAR
2	Gibbs‐Ward (2005) USA	Nutrition	Self‐care	−	U	U	−	−	−	−	+	U	+	LOW
3	Heaven (2013) UK	Nutrition	Self‐care	−	U	U	+	+	−	U	U	U	−	LOW
4	Mentes (2006) USA	Nutrition	Self‐care	−	U	U	U	+	U	U	U	U	U	LOW
5	Palacios‐Cena (2013) Spain	Nutrition	Self‐care	−	U	U	+	+	U	U	+	U	+	LOW
6	Roberts (2014) UK	Nutrition	Self‐care	−	−	U	+	−	−	−	+	U	+	LOW
7	Robison (2015) UK	Nutrition	Self‐care	−	−	U	+	+	+	+	+	U	+	HIGH
8	Schell (1999) USA	Nutrition	Self‐care	−	U	U	U	−	U	U	U	U	U	LOW
9	Sidenvall (1994) Sweden	Nutrition	Self‐care	−	+	U	+	−	−	U	+	U	U	LOW
10	Steele (2007) Canada	Nutrition	Self‐care	+	−	+	+	+	−	U	U	U	+	UNCLEAR
11	Xia (2006) Australia	Nutrition	Self‐care	−	U	U	+	+	−	+	+	U	+	UNCLEAR
12	Sjögren Forss (2018) Sweden	Nutrition	Self‐care	+	−	+	+	−	−	+	+	+	+	HIGH
13	Holst (2011) UK	Nutrition	Nutritional support	−	U	U	U	+	−	U	+	U	+	LOW
14	Kayser‐Jones (1999) USA	Nutrition	Nutritional support	−	U	U	+	−	U	U	+	U	−	LOW
15	Pasman (2003) Netherlands	Nutrition	Feeding protocols	+	−	U	+	−	U	U	+	U	+	LOW
16	Brady (2016) UK	Elimination	Multi‐component incontinence management	−	U	U	+	−	−	U	+	U	+	LOW
17	French (2016) UK	Elimination	Multi‐component incontinence management	−	+	+	+	+	−	+	+	U	+	HIGH
18	Thomas (2014) UK	Elimination	Multi‐component incontinence management	−	+	+	+	+	−	+	+	U	+	HIGH
19	Gibson (2018) UK	Elimination	Multi‐component incontinence management	+	−	−	−	+	−	−	+	+	+	LOW
20	Boltz (2011) USA	Mobility	Promoting independent mobility	−	+	+	+	−	+	+	+	U	+	HIGH
21	Bourret (2002) USA	Mobility	Promoting independent mobility	+	−	+	+	−	−	+	+	U	+	HIGH
22	Doherty‐King (2013) USA	Mobility	Promoting independent mobility	−	+	U	+	+	−	U	+	U	+	LOW
23	Kindblom‐Rising (2007) Sweden	Mobility	Promoting independent mobility	−	+	U	+	−	−	+	+	U	+	UNCLEAR
24	Kneafsey (2013) UK	Mobility	Promoting independent mobility	−	+	U	+	−	+	+	+	U	+	HIGH
25	Taylor (2014‐1) Australia	Mobility	Promoting independent mobility	−	+	+	+	+	U	U	+	U	+	HIGH
26	Taylor (2014‐2) Australia	Mobility	Promoting independent mobility	−	+	+	+	−	U	U	+	U	+	UNCLEAR
27	Taylor (2014‐3) Australia	Mobility	Promoting independent mobility	−	+	+	+	+	+	+	+	U	+	HIGH
28	Barber (2015) Australia	Mobility	Risk reduction	+	U	+	+	−	+	−	+	U	+	HIGH
29	Bradway (2010) USA	Hygiene	Cleaning people	−	+	U	U	−	U	U	U	U	+	LOW
30	Coyer (2011) USA	Hygiene	Cleaning people	+	+	+	+	+	+	U	+	U	+	HIGH
31	Gaspard (2012) Canada	Hygiene	Cleaning people	+	+	+	+	−	−	U	+	U	+	HIGH
32	Gibb (1990) Australia	Hygiene	Cleaning people	+	+	+	U	−	U	U	U	−	U	LOW
33	Jackson (2014) USA	Hygiene	Cleaning people	−	+	U	+	−	−	U	+	U	U	LOW
34	Lezzoni (2012) USA	Hygiene	Cleaning people	−	+	+	*N*/A	−	*N*/A	*N*/A	*N*/A	*N*/A	*N*/A	UNCLEAR
35	Miller (1997) USA	Hygiene	Cleaning people	+	U	U	+	−	−	U	+	U	+	LOW
36	Chalmers (1996) Australia	Hygiene	Oral health	−	U	U	+	−	−	−	U	U	U	LOW
37	De Visschere (2015) Belgium	Hygiene	Oral health	−	U	U	U	−	U	+	+	U	+	LOW
38	Sonde (2011) Sweden	Hygiene	Oral health	−	U	U	+	−	U	U	U	−	+	LOW
39	Wardh (2000) Sweden	Hygiene	Oral health	−	+	+	U	−	+	+	U	U	+	HIGH
40	Jensen (2013) Denmark	Hygiene	Self‐care	−	+	U	+	+	U	+	+	U	+	HIGH
41	Lomborg (2005) Denmark	Hygiene	Self‐care	+	+	+	+	+	U	+	+	U	+	HIGH
42	Lomborg (2008) Denmark	Hygiene	Self‐care	−	U	U	+	−	U	U	+	U	+	LOW
43	Edvardsson (2009) Sweden	Hygiene	Clothing	−	+	U	U	−	U	U	+	U	+	LOW
44	Kalish (2012) USA	ONB	Care as usual focusing on 2 or more essential care areas	−	U	U	U	−	−	U	−	U	−	LOW
45	Kitson (2013b) Australia	ONB	Care as usual focusing on 2 or more essential care areas	+	+	+	+	−	+	+	+	U	+	HIGH
46	Wolf (1993) Not given	ONB	Care as usual	+	+	+	U	−	U	U	U	U	U	LOW
47	Lafrenière (2017) Canada	ONB	Care as usual focusing on 2 or more essential care areas	+	+	+	+	−	+	+	+	+	+	HIGH

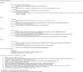

We identified 16 high‐quality studies, in terms of their methodological conduct (a clear research question, clear theoretical underpinning, an appropriate study design to answer the research questions and adequately reported data collection or analysis) that reported on an experimental study into a new method of nursing care with strong theoretical underpinnings and/or aimed to reflect opinions on implementation of nursing care actions. Within this 16, we identified six conceptually rich papers (Boltz, Capezuti, & Shabbat, 2011; French et al., 2016; Jensen, Vedelo, & Lomborg, 2013; Lomborg, Bjorn, Dahl, & Kirkevold, 2005; Robison et al., 2014; Thomas et al., 2014) that made a greater contribution to our understanding of the context of high‐quality fundamental care. These six papers then formed our preliminary analytical framework, to which the data in the remaining high‐quality papers were added (Malpass et al., 2009).

### 3.4 Scope of the high‐quality papers used in our qualitative synthesis

Of the 16 high‐quality studies, study designs were reported as grounded theory (3), ethnography (2), phenomenology (1), soft systems approach (1) and interpretative description (1), or reported as content analysis (3), thematic analysis (2), or framework analysis (1) or did not name the methodological orientation (2). Papers reported qualitative data for observational studies (*n* = 12) (Boltz et al., 2011; Bourret, Bernick, Cott, & Kontos, 2002; Coyer, O'Sullivan, & Cadman, 2011; Gaspard & Cox, 2012; Kitson, et al., 2013b; Kneafsey, Clifford, & Greenfield, 2013; Lafreniére, Folch, & Bèdard, 2017; Lomborg et al., 2005; Sjögren Forss, Nilsson, & Borglin, 2018; Taylor, Sims, & Haines, 2014a, 2014b; Wardh, Hallberg, Berggren, Andersson, & Sorensen, 2000) and experimental studies (*n* = 4) where new practices were introduced (French et al., 2016; Jensen et al., 2013; Robison et al., 2014; Thomas et al., 2014). Of these, two papers included patient data about a new nursing care method. Nine studies included patient's perspectives of experience of care (Bourret et al., 2002; Jensen et al., 2013; Kitson et al., 2013b; Lafreniére et al., 2017; Lomborg et al., 2005; Robison et al., 2014; Sjögren Forss et al., 2018; Taylor, Sims, & Haines, 2014b; Thomas et al., 2014).

Data were collected using interviews only (7), focus groups only (5) and interviews and focus groups (2), or observations and interviews (2). Data were collected from nurses only (8), patients or residents only (5) and nurses and patents (3). Five studies were about hygiene (cleaning people × 2, oral hygiene × 1 and assisted body care × 2), five mobility (promoting independent mobility × 4, mobility maintenance × 1), two elimination (multi‐component incontinence management), two nutrition (mealtime assistance) and two addressed more than two fundamentals of care (Table 2). Ten studies were conducted in hospital and six in care homes.

## 4 SYNTHESIS OF NURSE BEHAVIOURS WITHIN EACH ESSENTIAL CARE AREA

In this section, the two high‐quality papers that described more than one care area (Kitson, Conroy, et al., 2013; Lafreniére et al., 2017) are discussed in the relevant care sections.

### 4.1 Hygiene

Within the five high‐quality studies about hygiene, findings indicated that nursing behaviours should include explanation of the content and purpose of hygiene care activities and should be tailored where possible to individual patients (Coyer et al., 2011; Gaspard & Cox, 2012; Kitson et al., 2013b; Wardh et al., 2000), such as considering patient wishes to use their own toiletries (Coyer et al., 2011). Patients recognised the impact of feeling clean on well‐being and integrity but reported the difficult balance between preservation and threats to integrity when receiving body care. Patients reported feeling part of a collaboration with nurses to achieve body cleanliness whilst minimising discomfort, and this helped to legitimise patients asking for and receiving assistance (Jensen et al., 2013; Lomborg et al., 2005).

### 4.2 Mobility

Patients reported valuing mobility and independence (Boltz et al., 2011; Bourret et al., 2002; Kitson et al., 2013b; Lafreniére et al., 2017; Taylor, Sims, & Haines, 2014a; Taylor et al., 2014b) and being assisted and encouraged to move according to abilities (Lafreniére et al., 2017). Patients appreciated actions to prevent falls (Lafreniére et al., 2017). Nurses noticed patients associating self‐worth with mobility (Bourret et al., 2002). Nurses considered effective strategies to promote independent mobility that involved providing encouragement, setting specific and achievable goals with patients, using appropriate mobility aids, pain relief prior to activities, developing flexible care plans with patients and adjusting these as patients or residents mobility improved (Boltz et al., 2011; Bourret et al., 2002; Kitson, Conroy, et al., 2013; Lafreniére et al., 2017; Taylor et al., 2014a). Other studies showed nurses paid limited attention to patients' rehabilitation goals but instead were concerned with “care to keep safe” (Kneafsey et al., 2013) and prevention of potential problems including falls (Kneafsey et al., 2013; Lafreniére et al., 2017; Taylor et al., 2014a).

### 4.3 Elimination

Both high‐quality elimination studies focussed on whether and how a new urinary incontinence rehabilitation and management protocol could become routine practice. Nurses reported challenges at the start due to a culture of nursing practice that encouraged urine containment rather than rehabilitation of incontinence. Nurses overcame difficulties and became enthused by working on a collective goal to rehabilitate patients. Nurses later recognised the benefits of improving incontinence for patients and the potential for reduction in their incontinence care workload (French et al., 2016; Thomas et al., 2014). One study suggested that patients with stroke preferred nurses who demonstrated sensitivity and provided full explanations about the process of using incontinence aids (Kitson et al., 2013b), and another indicated patients want assistance getting to the toilet to prevent incontinence (Lafreniére et al., 2017).

### 4.4 Nutrition

One high‐quality paper about nutrition described the views of nurses, patients and relatives about the introduction of trained volunteers to provide mealtime assistance to elderly people in an acute medical ward (Robison et al., 2014). Other studies reported patients, residents, nurses and relatives appreciating the time nurses (or volunteers) were able to give support residents to eat (Robison et al., 2014; Sjögren Forss et al., 2018) such as in preparing patients for eating, opening containers, offering and explaining options of what and when to eat, and providing assistance and encouragement to eat. Residents wished for more autonomy in choosing their own meals and when and where they could eat (Lafreniére et al., 2017; Sjögren Forss et al., 2018).

## 5 CONCEPTUAL SYNTHESIS OF PAPERS ACROSS ESSENTIAL CARE AREAS

We derived concepts from substantive themes describing essential nursing care across the four care domains, identified in the six conceptually rich articles and the remaining eight high‐quality articles. The three conceptual themes are key factors influencing high‐quality care and its implementation in practice: nurse leadership, partnerships with patients and organisational practices (Table 3).

**Table 3:** Translation of themes into concepts

Overarching conceptual themes	Substantive themes: Concepts derived from author themes	Interpretation of author themes of facilitators and barriers to essential nursing care	Papers that include the constructs (with papers that were conceptually rich in bold)
Nurse Leadership	Generating buy‐in	Leaders are involved in work to generate enthusiasm and support for the intervention by helping them to see the importance and changes as worthwhile for both patients and nurses	**Boltz; French; Robison; Thomas**
	Nurse learning and competency	Leaders supported nurses to gain relevant knowledge and skills (French, Thomas, Robison) by assessing competencies, offering feedback and training (Thomas, Taylor 2014‐1) nurses required training about techniques to care for patients, understanding the purpose and targets for care, organisational “priorities” and “role responsibilities”	**Boltz; French; Robison; Thomas**; Gaspar; Kitson 2013b; Kneafsey; Taylor 2014a, b; Wardh
	Defining and enabling nurse caring roles	Agreed procedures for structured care endorsed by effective organisation of staff with clear role responsibilities and facilitation and empowerment of staff to make decisions	**Boltz; French; Jensen; Robison; Thomas;** Bourret; Coyer; Gaspard; Kitson 2013b; Kneafsey; Taylor 2014 a,b; Wardh
	Teamworking	Where essential care is organised well, and nurses are given nurses collaborate and co‐ordinate care work between themselves and have a ‘positive working relationship’. Opportunities for teamworking with other health care professionals are welcomed such as participation in interdisciplinary meetings	**Boltz; French; Robison, Thomas,** Coyer; Bourret; Gaspard; Kneafsey; Taylor 2014‐b; Wardh
Partnerships with patients	Patient centred care	Care that takes into account the health, capabilities, needs and preferences of the patient whilst “showing kindness.” Trust is developed and care decisions are discussed with the patient and decided taking into account the patient's limitations Patients are encouraged to engage in their own care activities where possible	**Boltz; French; Jensen; Lomborg;**, Bourret; Coyer; Gaspard; Kitson 2013b, Kneafsey; Robison; Taylor 2014a
	Continuity of care	Care delivered in an environment where patient care is experienced as consistent by patients and is agreed, standardised and shared between staff members and teams	**Boltz; French; Robison; Thomas;** Bourret,; Gaspard,; Kitson 2013b, Kneafey,; Taylor 2014a, ;Wardh
	Management of patient expectations	Explaining to patients of the normal expectations of care procedures with the option of some flexibility and the expectation for patients to be involved in their own care and recovery	**Boltz; French; Jensen; Lomborg;** Bourret; Kitson; Kneafsey; Wardh
Organisational practices	Staffing and time constraints	Perceptions of lack of time to perform care activities can be improved by organisation of resources and role responsibilities and increasing the prioritisation of care activities, and supporting change expectations with appropriate resources	**Boltz; French; Jensen; Robison; Thomas;** Bourret; Coyer; Gaspard; Kitson 2013b; Kneafsey**;** Taylor 2014a
	Policy and procedure	Organisational policy aligned to the nursing care objectives helps endorse care activities but can impact negatively the ability of nurses to perform care activities if they are not aligned. The nursing physical environmental and equipment can reflect organisational policy and can be a barrier to essential care on both a practical level and on a cultural	**Boltz; French; Robison;** Bourret; Coyer; Gaspard; Kneafey; Kitson 2013b; Thomas, Taylor 2014a,b; Wardh

### 5.1 Nurse leadership

Nurse leadership is about the necessary actions and influence of people to inspire teach and support nurses and nurse teams to perform new or consistently high‐quality nursing care practices. Strong leaders were able to “counteract established perceptions” (French et al., 2016) and make judgements on nursing care plan changes that others would follow (Thomas et al., 2014) and were seen as influencing change by encouraging others and pushing practice forward (Taylor et al., 2014b). People that had influence on nurses were senior nurses, physiotherapists (Kneafsey et al., 2013; Taylor et al., 2014b), research nurses (French et al., 2016) and experienced nurse colleagues or peer leaders (Gaspard & Cox, 2012; Taylor et al., 2014b; Thomas et al., 2014). We derived four concepts from author themes about nurse leaders**'** actions that were associated with nurses consistently performing essential nursing care, these were “generating buy‐in,” “nurse learning and competency,” “defining and enabling nurse roles” and “teamworking.”

#### 5.1.1 Generating buy‐in

Buy‐in relates to whole nursing team commitment (Boltz et al., 2011) and enthusiasm to commit and act on a proposed change in practice. Whole team buy‐in facilitates a “standardised consistent approach” (Robison et al., 2014 p141) by all members, including administrative staff (Boltz et al., 2011). Studies reported buy‐in and staff commitment when “key people” (French et al., 2016 p1398) led change by advocating and demonstrating the importance and advantages of the proposed care practices (Boltz et al., 2011; French et al., 2016; Thomas et al., 2014).

Buy‐in was reinforced by gaining experience. In some intervention studies, initially nurses did not have full belief in proposed changes and were sceptical about making changes, but once nurses were encouraged and supported to start implementing changes and experienced positive results, they were more willing to engage with new practices (Robison et al., 2014; Thomas et al., 2014). For example, nurses appreciated being formally shown how care practices were important and of benefit to patients (Boltz et al., 2011; French et al., 2016), seeing an increase in their “therapeutic role” (French et al., 2016 p1398) and seeing how practices would “reduce workload in the long run” (French et al., 2016 p1399).

Buy‐in was evident when nursing practices were linked to a clear priority in the organisation (Coyer et al., 2011; French et al., 2016) and where nursing priorities were visible in organisations' targets and procedures (Boltz et al., 2011). Several papers recommended that nurses should be explained how nursing practices relate to institutional targets or priorities (Boltz et al., 2011; Coyer et al., 2011; French et al., 2016; Kitson et al., 2013b; Kneafsey et al., 2013; Robison et al., 2014; Taylor et al., 2014b; Thomas et al., 2014; Wardh et al., 2000).

#### 5.1.2 Nurse learning and competency

Nurses considered a lack of knowledge, skills and confidence in delivering essential patient care as barriers to high‐quality care (Boltz et al., 2011; Kneafsey et al., 2013; Wardh et al., 2000). For example, care could be inconsistent when individual nurses lacked essential skills and training (Robison et al., 2014; Thomas et al., 2014; Wardh et al., 2000). Information about effective protocols and procedures of care and examples of best practices was not standardised (Boltz et al., 2011; Coyer et al., 2011) but needed to be arranged and communicated effectively between all involved in care (Boltz et al., 2011; French et al., 2016; Robison et al., 2014; Thomas et al., 2014). Nurses reported feeling powerless in care‐related decision‐making (Kneafsey et al., 2013; Robison et al., 2014; Taylor et al., 2014b), such as not knowing how to prioritise when many patients needed help (Kneafsey et al., 2013). Nurses relied on their generalist knowledge (Thomas et al., 2014; Wardh et al., 2000) rather than taught knowledge (Wardh et al., 2000). Only three studies included formal essential care training (French et al., 2016; Robison et al., 2014; Thomas et al., 2014). Those who had received training felt better prepared and aware of patients' specific care needs (Wardh et al., 2000).

Nurses reported feeling able to incorporate nursing care initiatives into their practice when time had been dedicated to training and support to learn (French et al., 2016; Robison et al., 2014; Thomas et al., 2014). Competence was evident when training was well supported and structured, but learning was also led by peer leaders who offered informal feedback and training, supervision and support to less competent or less experienced nurses (Taylor et al., 2014a; Thomas et al., 2014; Wardh et al., 2000).

Nurses reported a need for improved skills and understanding to instil confidence in delivering necessary care (Robison et al., 2014). Nurses reported benefitting from improved understanding of the purpose and importance of care procedures (Robison et al., 2014; Thomas et al., 2014) with agreed team goals (Boltz et al., 2011; French et al., 2016; Wardh et al., 2000).

#### 5.1.3 Defining and enabling nurse caring roles

Confusion over allocation of work and division of labour could disrupt engagement with agreed care protocols (Thomas et al., 2014; Wardh et al., 2000). When care responsibilities were not well‐understood nurses described lack of autonomy in prioritising fundamental care over other competing nursing tasks (Coyer et al., 2011; Robison et al., 2014; Thomas et al., 2014). Conversely, nurses reported that good management of existing staff resources with clear role responsibilities was enabling factors for staff to work effectively on agreed care priorities (Boltz et al., 2011; Bourret et al., 2002; Coyer et al., 2011; Robison et al., 2014; Thomas et al., 2014). Nurses wanted clarity on what was expected of them, their tasks and required actions, and shared duties (Boltz et al., 2011; French et al., 2016; Gaspard & Cox, 2012; Robison et al., 2014; Taylor et al., 2014a, 2014b; Thomas et al., 2014; Wardh et al., 2000). Nurses were able to work effectively when supported by leadership to help organise care activities, and to consider how and when care tasks were to be performed (Bourret et al., 2002; Coyer et al., 2011; Kneafsey et al., 2013; Robison et al., 2014; Thomas et al., 2014; Wardh et al., 2000).

When empowered, nurses wanted to take responsibility for the details of care delivery (Boltz et al., 2011; French et al., 2016; Gaspard & Cox, 2012; Kneafsey et al., 2013; Robison et al., 2014; Taylor et al., 2014a; Thomas et al., 2014). For example, some nurses were confident in knowing when changes in residents' mobility status had occurred and this confidence extended to them making judgements regarding care plan changes (Taylor et al., 2014a). However, without clear responsibilities there could be confusion and uncertainty about making even relatively minor decisions, such as which incontinence aids to use (Taylor et al., 2014a), and this created frustration for nurses (Kneafsey et al., 2013). Nurse's engagement with required nursing care practices was linked to nurses' belief that they could voice concerns to senior colleagues about current practices and could help to improve procedures (Coyer et al., 2011).

#### 5.1.4 Teamworking

Teamwork occurred when staff worked with each other to co‐ordinate their efforts and find meaningful ways to “develop and embed new practice” (Thomas et al., 2014 p1315) and where there were expectations that decisions would be supported by all members of the team (Gaspard & Cox, 2012). Teamwork could involve nurses working with other healthcare professionals and was more likely when practices were prioritised by wider leadership (Boltz et al., 2011), for example where written plans were structured and formal (Robison et al., 2014) with accountability for care by all team members (Boltz et al., 2011; Robison et al., 2014; Thomas et al., 2014).

Working together and positive working relationships with the team leader were considered important for successful care implementation (Boltz et al., 2011; Bourret et al., 2002; Gaspard & Cox, 2012; Kneafsey et al., 2013; Taylor et al., 2014a; Wardh et al., 2000). For example, nurses needed to effectively and routinely share information about the care provided and decisions about care. There was evidence for teamworking to communicate clear and easily accessible information about patient care (French et al., 2016) such as using symbols on a whiteboard or in a patient's handover chart (French et al., 2016; Taylor et al., 2014b). Another example of teamworking was in whole team discussions to agree on care actions to be taken (Boltz et al., 2011; Bourret et al., 2002; Robison et al., 2014; Taylor et al., 2014a, 2014b; Thomas et al., 2014). Although working with the wider interdisciplinary team to complete essential care was thought to be useful for patients (Boltz et al., 2011; Bourret et al., 2002; Gaspard & Cox, 2012; Kneafsey et al., 2013), only one study described an example, where interdisciplinary teams visited patients together in “interdisciplinary rounds” (Boltz et al., 2011 p220). A perceived lack of teamwork was reported as a source of stress for nurses (Boltz et al., 2011) and when care activities were ad hoc rather than planned and not co‐ordinated between staff (Kneafsey et al., 2013; Wardh et al., 2000).

### 5.2 Partnerships with patients

Partnerships with patients concern the specific work by nurses with patients to optimise patients' satisfaction with care. Many papers reported nursing care with a rehabilitative element promoting patient independence and discussed the work required by nurses and nurse teams to develop a collaborative partnership with patients to meet patient needs (Boltz et al., 2011; Bourret et al., 2002; Coyer et al., 2011; French et al., 2016; Gaspard & Cox, 2012; Jensen et al., 2013; Kitson et al., 2013b; Kneafsey et al., 2013; Lomborg et al., 2005; Robison et al., 2014; Taylor et al., 2014a). We derived three concepts about partnerships with patients from author themes, and these were “person‐centred care,” “continuity of care” and “management of patient expectations.”

#### 5.2.1 Person‐centred care

The promotion of self‐care with consideration of the patients' needs was a favoured approach mentioned in all care areas. Person‐centred care required engagement with and involvement of patients as participants in their own care (Boltz et al., 2011; Bourret et al., 2002; French et al., 2016; Jensen et al., 2013; Robison et al., 2014; Taylor et al., 2014b) rather than nurses making assumptions about patients' care needs and “doing” for them (Boltz et al., 2011 p219). Nurses understood time was needed to attend to needs and not to rush (Lomborg et al., 2005) and to take into account the patients' current condition, their abilities and their fears (Bourret et al., 2002; Coyer et al., 2011; Lomborg et al., 2005; Taylor et al., 2014a), with goals for progression that were understood and considered to be achievable by the patient (Boltz et al., 2011; Jensen et al., 2013; Kitson et al., 2013b; Taylor et al., 2014a; Thomas et al., 2014). Person‐centred care had a structure with flexibility. Patients were offered options of how necessary care could be undertaken (Bourret et al., 2002; Jensen et al., 2013; Kitson et al., 2013b; Robison et al., 2014; Taylor et al., 2014b) with an opportunity to adjust care activities according to patients changing needs with changes in health (Boltz et al., 2011; French et al., 2016; Jensen et al., 2013; Thomas et al., 2014).

Patients valued nurse compassion in dealing with their essential care needs (Boltz et al., 2011; Jensen et al., 2013; Kitson et al., 2013b). This was reflected in nurses being “friendly,” “nice” and “listening” (Jensen et al., 2013 p1010), demonstrating kindness, such as using comforting touch and focussing on the patient rather than on tasks (Coyer et al., 2011; Gaspard & Cox, 2012; Kneafsey et al., 2013; Taylor et al., 2014b; Wardh et al., 2000). A considerate approach was reported to build trust and understanding between nurse and patient (Bourret et al., 2002) and lead to collaboration and honest mutual information sharing in both directions between nurse and patient (Gaspard & Cox, 2012; Jensen et al., 2013; Kitson et al., 2013b). Studies also report that patients recognised lack of availability of nurses and negative reactions to requests for assistance affected their ability to maintain good spirits value nursing being available and receptive to requests of help (Boltz et al., 2011; Bourret et al., 2002; French et al., 2016; Jensen et al., 2013; Thomas et al., 2014).

#### 5.2.2 Continuity of care

Continuity of care was perceived to be an indicator of quality by both patients and nurses (French et al., 2016; Kitson et al., 2013b; Taylor et al., 2014a; Thomas et al., 2014). It refers to care delivered consistently between members of staff towards patients (Kitson et al., 2013b; Taylor et al., 2014a). As patients can be cared for by a number of individuals during a hospital stay or as a care home resident, nurses recommended that there is team alignment to jointly agreed care paths (Gaspard & Cox, 2012) with clear targets and objectives (Boltz et al., 2011; French et al., 2016; Kneafsey et al., 2013; Robison et al., 2014) and personalised care plans are recorded and shared during shift handover (Boltz et al., 2011; Gaspard & Cox, 2012; Jensen et al., 2013; Robison et al., 2014; Thomas et al., 2014; Wardh et al., 2000).

#### 5.2.3 Management of patient expectations

Working with patients and relatives to explain the type of nursing care they would expect to receive was seen as an important step to gaining co‐operation with self‐care or accepting care support (Boltz et al., 2011; French et al., 2016; Jensen et al., 2013; Kitson et al., 2013b; Lomborg et al., 2005; Robison et al., 2014; Wardh et al., 2000), especially in care activities with a rehabilitation element such as enhancing physical function, incontinence training and feeding (Boltz et al., 2011; French et al., 2016; Kitson et al., 2013b; Kneafsey et al., 2013; Thomas et al., 2014; Wardh et al., 2000). Managing patient expectations involved nurses explaining the details of the care, written agreements, the role of any other health professionals involved in achieving recovery objectives (Kitson et al., 2013b) and helping patients to understand their own capabilities (Jensen et al., 2013; Thomas et al., 2014). Patient's views of their own independence expectations were perceived by nurses to be influenced by relatives and could impede the promotion of independence. Nurses believed that relatives expected or encouraged their loved one to do little, or to rest and be cared for rather than participate in their own care (Boltz et al., 2011; Lomborg et al., 2005). Involving patients and family members in conversations about care (Boltz et al., 2011; Robison et al., 2014) helped to reinforce the potential impact of elements of planned care activity (Kitson et al., 2013b) and highlight the potential risks of essential care needs not being met (Boltz et al., 2011).

### 5.3 Organisational practices

The conceptual theme “organisational practices” relates to the influence of the nurses' working environment on assisting, helping or obstructing essential nursing care. Some embedded nursing care practices were cited by authors as a hindrance to making changes to improve nursing care. Usual nurse care practices were described as part of the culture within institutions. Introduction of new or adjusted practices required planning and support to fit with existing procedures (Robison et al., 2014; Taylor et al., 2014b; Thomas et al., 2014). Two key concepts about the influence of the organisational practices on nursing practice were derived from author themes. These were “staffing and time constraints” and “policy and procedure.”

#### 5.3.1 Staffing and time constraints

Lack of time to deal with all the necessary care activities was common reason nurses and patients gave for not consistently addressing patients' fundamental care needs. Nurses reported lack of time to perform care responsibilities or that care activities were time‐consuming due to other more important “competing priorities” (Kneafsey et al., 2013; Robison et al., 2014; Thomas et al., 2014). This could be compounded by a perception of a lack of resources or designated staff to perform specific care duties (Boltz et al., 2011; French et al., 2016; Robison et al., 2014; Thomas et al., 2014). Care duties in several care interventions were seen as additional work; however, having extra staff did not mean that workload was perceived to be reduced (French et al., 2016). Care‐related workload stress was present when there was a “lack of direct patient care time” (Kneafsey et al., 2013 p1625) and a lack of task management and organisation, structure and planning (Taylor et al., 2014b).

#### 5.3.2 Policy and procedure

Some studies reported that organisational policies did not prioritise essential nursing care (Coyer et al., 2011; Robison et al., 2014; Thomas et al., 2014). Nurses felt that care strategies were important but had been underestimated and not supported. Support for nursing care as key priorities was seen to help facilitate changes, but where nursing practices appeared to conflict with current organisational policies, attempts to optimise nursing care were hampered (Coyer et al., 2011; Robison et al., 2014; Thomas et al., 2014). For example, nurses struggled to follow a prompted voiding protocol for patients who were incontinent within a nursing culture of containing urine and faeces using catheters and incontinence pads rather than rehabilitating patients to help reduce incontinence (Thomas et al., 2014). Similarly, nurses struggled to encourage rehabilitative mobility when nurses were more focussed on minimising risk of falls (Boltz et al., 2011; Bourret et al., 2002; Kneafsey et al., 2013). Nurses reported working in ways that were not consistent with their beliefs of what constituted quality care because they were not empowered to challenge the institution (Coyer et al., 2011). Consequently, nursing care activities were considered to be easier when care activities were endorsed by management (Coyer et al., 2011; Kneafsey et al., 2013; Robison et al., 2014; Thomas et al., 2014; Wardh et al., 2000) and specifically included in organisational policy and procedure (Coyer et al., 2011) with targets and reporting (Boltz et al., 2011; Thomas et al., 2014). Where there was “synergy between other initiatives,” care practices were thought to be easier to embed (Robison et al., 2014).

Some aspects of the nursing environment and use of nursing equipment were reported to impede nursing care objectives to optimise patients' recovery and independence. These were considered to be reinforced by the culture of care within the organisation (Bourret et al., 2002; Coyer et al., 2011; Gaspard & Cox, 2012). Physical restrictions included Foley catheters as “tethers” (Boltz et al., 2011), the use of bedside rails, restraints and imposed restrictions on space to walk or inadequate lighting (Boltz et al., 2011; Bourret et al., 2002). In one study, a “minimal handling” approach disempowered nurses to mobilise patients without the input from a physiotherapist (Kneafsey et al., 2013 p1626). Conversely, raised toilet seats, adequate flooring, having access to gardens and access to appropriate equipment (Boltz et al., 2011; Bourret et al., 2002; Kneafsey et al., 2013) were examples of environmental factors that facilitated mobility. In some reports, identification and minimisation of environmental or procedural restraints could assist nurses in their care delivery (Bourret et al., 2002; Coyer et al., 2011; Gaspard & Cox, 2012).

## 6 DISCUSSION

**Figure 2:**
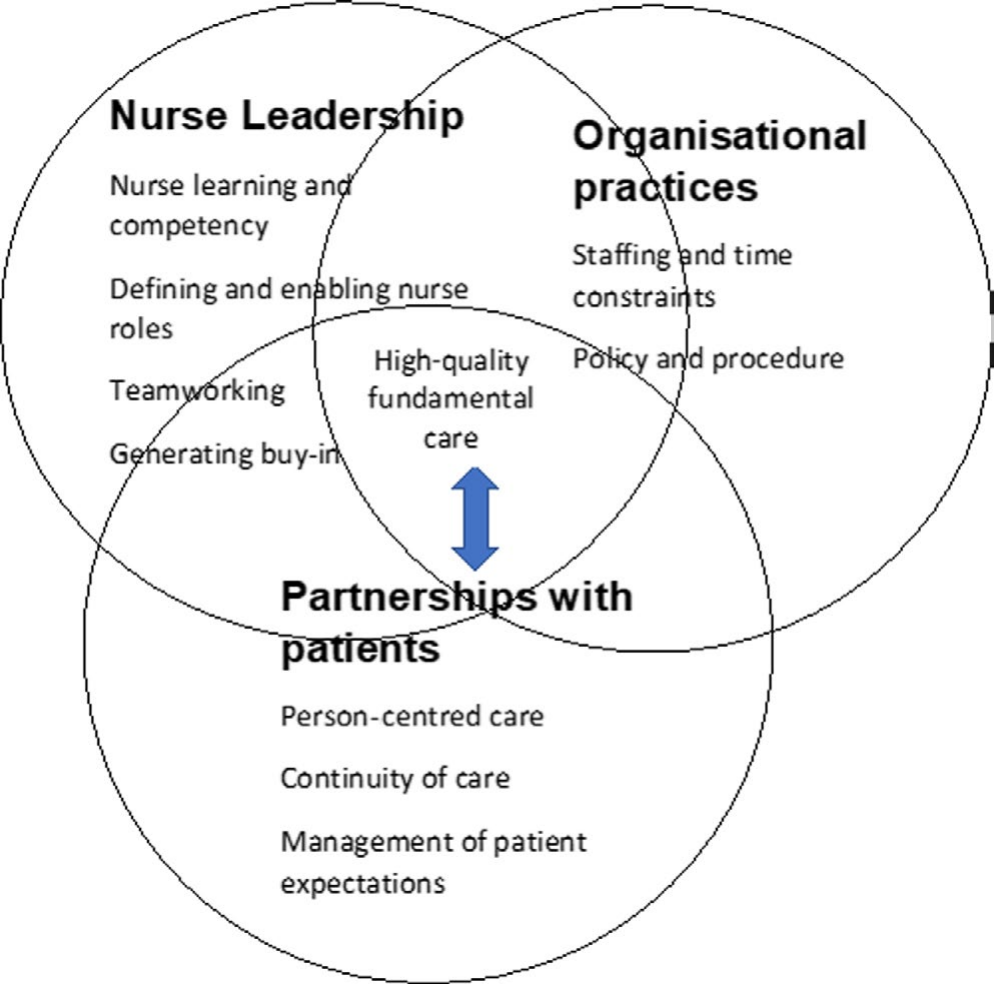
Diagram of Conceptual themes

Our synthesis of reports from 16 qualitative studies demonstrates that experiences of nurses and patients receiving or delivering high‐quality fundamental care can be interpreted in three conceptual themes: (a) nurse Leadership, (b) partnerships with patients and (c) organisational practices (Figure 2). Nurse leadership is the endorsement, direction, guidance and support from people with influence that is necessary to drive nurses to embed essential care activities in their usual practice. Partnership with patients is the nursing work necessary to ensure patients have the opportunity to influence and be involved in the content and method of their care. Organisational practices are standard processes that are fostered by written policies or historical procedures and influenced by organisational targets which have an impact on the methods, nature and culture of nursing care activities. These three concepts together are essential to the provision of fundamental care.

Across the 47 studies, we found most studies were about hygiene and mobility, and fewer studies were about elimination and nutrition. Qualitative data typically focussed on observations of usual care highlighting missing or incomplete care and few described experimental studies about improving essential nursing care interventions. The 16 high‐quality studies showed the nursing behaviours addressing essential care needs involve assisting patients to be as independent as possible in their hygiene, mobility, toileting, eating and hydration by considering their abilities. The management of comfort and distress is achieved through mutual agreement with patients on a strategy for care activities through clear explanation and discussions about patient choice achieved through strong relational skills. We found common support for assisting patients to self‐care by increasing their self‐awareness, using target‐based goal setting, and only offering assistance when it was needed.

Our findings indicate that nurses and patients believe that managing patient's expectations of care and educating patients about what to expect of care could encourage engagement in their own recovery. Working with patients to encourage them to engage with self‐care has been shown to reduce length of stay (Dutton, Daugherty, Mason, & McGrath, 2014; Gustafsson et al., 2012; Jones et al., 2012; Paton et al., 2014).

We have demonstrated that nursing care which considers the patient with unique needs by offering choice and flexibility is valued by patients. Patients want to be involved in their own care. However, this synthesis has found that to deliver high‐quality nursing care it is not enough to explore and identify the effective components of nurse–patient interactions.

The detailed data in the six conceptually rich papers enabled us to identify three conceptual themes: nurse leadership, partnerships with patients and organisational practices, which we interrogated and tested using data from the wider pool of 16 high‐quality papers. These additional data further broadened our understanding of these three concepts as essential to the provision of the fundamentals of nursing care and key to any future intervention.

All three need to work together to allow nurses to meet patients care needs. Strong leaders are required who effectively manage nurses' roles and their time to allow for care duties. Leaders need to educate, encourage and enable nurses to work together to facilitate improvements to care practices and to ensure that patient care is person‐centred and follows best practice. Although nurses can work together to resolve some of the barriers to caring, especially in the presence of strong leadership, the quality of care is likely to be compromised or unsustainable when there is a lack of wider organisational support. The conceptual theme “organisational practices” highlights the difficulties nurses have in the workaround “partnerships with patients” that is—the essential nursing care work and interactions with patients, when there is an absence of organisational targets or policies for fundamental nursing care activities. Meeting patients care needs are easier for nurses and their teams when the overarching organisation removes as many barriers concerning existing policies and procedures that may hinder nursing care practices and is shown to prioritise caring activities so it is considered equal to rather than as competing with other priorities.

## 7 STRENGTHS AND LIMITATIONS

We have performed the first systematic qualitative synthesis of papers reporting qualitative data on fundamental nursing care in the key areas of hygiene, mobility elimination and nutrition. This is an important step forward to identifying areas which have implications for further research and practice. We have synthesised descriptions of experiences in high‐quality papers about fundamental nursing care and have presented evidence to show the key elements of nursing care practice, and evidence that wider contextual factors within the organisation need to be considered.

Our synthesis was the result of an extensive search and review of a large amount of data including the perspective of qualified and unqualified nurses, and patients in hospital and residents of care homes. Although our search was thorough, we may have missed some studies.

Few studies explored the impact of specific nursing behaviours on patients' experiences of care, for example there was no evidence about usual toileting preferences of people in hospital or care homes. In addition, most patients in the studies had very specific nursing care needs which may limit the generalisability of our findings. None of the studies reported patient and public involvement (PPI) strategies. PPI is considered a cornerstone of good quality healthcare research in the UK (National Institute of Health Research, 2012).

## 8 IMPLICATIONS

This new knowledge can be applied to the concept of Amalgamation of Marginal Gains by considering the three conceptual themes and the substantive themes as a framework. Patient representatives and nurses, including healthcare assistants and senior nurses should be involved in processes to identify areas to make small changes to patient care, to identify optimal ways to measure and monitor successes, and methods to feedback the results of care practices to all involved. There should be a clear organisational emphasis of the importance of nursing care practice determined by an agreed collective target reflecting an improvement in the quality of fundamental nursing care that represents the needs of all concerned (Pentecost et al., 2018); the patients, nurses, leaders and the wider organisation. When the target is understood and agreed the process of identification of small areas to make changes to achieve can follow.

Our findings have clinical implications for practice. Alongside our previous systematic review (Richards et al., 2018) and work to understand the practical application of Amalgamation of Marginal Gains (Pentecost et al., 2018), the findings will help us to develop a nursing care intervention that may have reasonable chance of operationalisation. We will include our qualitative findings to inform the development of an intervention to improve nursing care alongside additional work involving patients and nurses. The intervention will be tested in practice for feasibility and effectiveness.

## 9 CONCLUSIONS

Fundamental nursing care is crucial for the safe and effective care of people in hospitals and care homes. We undertook a review of the qualitative evidence to understand patients' and nurses' experiences of fundamental care to assist in the development of an intervention to improve the experience of care. Qualitative evidence about essential nursing care behaviours is often of poor quality. It is collected from studies in specific nursing contexts and does not link fundamental care behaviours to positive patient experiences. We have synthesised those studies that can best inform our nursing intervention and considered the findings to inform an intervention. Our synthesis indicates that to improve patient experience of care, strong leaders are required to clarify the objectives and targets of the care activities and to enthuse and support staff to embed consistent nursing care practices, nurses should work with individual patients to meet their care requirements and to encourage self‐care, and the overarching organisation needs to be actively supportive and to recognise the value of fundamental nursing care. All three areas may need to be addressed to improve the quality of fundamental nursing care, over and above carrying out the actual fundamental nursing care itself.

## 10 RELEVANCE TO CLINICAL PRACTICE

Qualitative evidence regarding fundamental nursing care is mostly of poor quality. There are few studies suitable to inform nursing practice. However, when planning a nursing intervention to improve patient experience of fundamental care three concepts may be important: effective nurse leadership, nurses**'** partnerships with patients and organisational prioritisation of fundamental nursing care. Nurse researches should conduct more rigorous mixed methods research to build knowledge of nursing care behaviours that may impact on patients**'** experiences of fundamental care.

## CONFLICT OF INTEREST

The authors declare that there are no conflicts of interest.
