# Supplementary material for: Patients' and nurses' experiences of fundamental nursing care: A systematic review and qualitative synthesis
Source: J Clin Nurs. 2019 Nov 19;29(11-12):1858–82. doi: 10.1111/jocn.15082 (PMC7319357; doi:10.1111/jocn.15082)
Supplement: Supplementary file 1 [file JOCN-29-1858-s001.docx]

**Appendix S1**

1. Exp Patients/
2. resident*.tw.
3. patient*.tw.
4. (recipient* adj care).tw.
5. or/1-4
6. exp Hospital/
7. exp Hospitalization/
8. Ward*.tw.
9. long term care/
10. care home*.tw.
11. Nursing home/ or “nursing home”*.tw.
12. hospital*.tw.
13. (aged adj2 (care or nursing or healthcare or residential) adj 2 (facility or facilities or home*))tw.
14. Assisted living facilities/ not Ambient assisted living/
15. exp Homes for the Aged/or “homes for the aged”.tw.
16. exp Housing for the Elderly/
17. exp Health Services for the Aged/
18. Care setting*.tw.
19. long-term care.tw.
20. ((Group or residential adj (care or unit or home* or facilities).tw.
21. (institution* or facility or facilities) adj5 (elder* or geriatric* or senior* or aged).tw.
22. ((retirement or residential) adj5 (house or home or accommodation).tw.
23. care or convalescent) adj (home? or cent* or facility or facilities).tw.
24. or/6-23
25. exp Nursing/
26. Nurs*.tw.
27. Nurse?.tw.
28. nursing.tw.
29. exp nurses/
30. exp nurse administrators/
31. exp nurse anesthetists/
32. exp nurse clinicians/
33. exp nurse midwives/
34. exp nurse practitioners/
35. exp nurses, international/
36. exp nurses, male
37. exp nursing staff/
38. exp nursing staff, hospital/
39. exp Nursing care/
40. patient care.tw.
41. exp patient care/
42. exp aftercare/
43. exp ambulatory care/
44. exp critical care/
45. exp custodial care/
46. exp day care/
47. exp "episode of care"/
48. exp life support care/
49. exp Long term care/
50. custodial care.tw.
51. exp night care/
52. exp palliative care/
53. exp patient positioning/
54. exp perinatal care/
55. exp perioperative care/
56. exp prenatal care/
57. exp preoperative care/
58. exp terminal care
59. or/25-58
60. Drink*.tw.
61. not alcohol.tw. or heavy adj drink*.tw.or excessive*adj drink*.tw.
62. water.tw.
63. fluid.tw.
64. hydrat*.tw.
65. meal*.tw.
66. undernutrition.tw.
67. under nutrition.tw.
68. nutrition.tw.
69. malnutrition.tw.
70. food.tw.
71. eat*.tw.
72. dining.tw.
73. feeding.tw.
74. breakfast*.tw.
75. dinner*.tw.
76. lunch*.tw.
77. tea.tw.
78. teatime.tw.
79. snack*.tw.
80. diet.tw.
81. dietary.tw.
82. or/60-81
83. Meta-Analysis as Topic/
84. meta analy$.tw.
85. metaanaly$.tw.
86. Meta-Analysis/
87. (systematic adj (review$1 or overview$1)).tw.
88. exp Review Literature as Topic/
89. or/83-88
90. cochrane.ab.
91. embase.ab.
92. (psychlit or psyclit).ab.
93. (psychinfo or psycinfo).ab.
94. (cinahl or cinhal).ab.
95. science citation index.ab.
96. bids.ab.
97. cancerlit.ab.
98. or/90-97
99. reference list$.ab.
100. bibliograph$.ab.
101. hand-search$.ab.
102. relevant journals.ab.
103. manual search$.ab.
104. or/99-103
105. selection criteria.ab.
106. data extraction.ab.
107. 105 or 106
108. Review/
109. 107 and 108
110. Comment/
111. Letter/
112. Editorial/
113. animal/
114. human/
115. 113 not (113 and 114)
116. or/110-112, 115
117. 89 or 98 or 104 or 109
118. 117 not 116
119. ("semi-structured" or semistructured or unstructured or informal or "in-depth" or indepth or "face-to-face" or structured or guide) adj3 (interview* or discussion* or questionnaire*).tw.
120. focus group* or qualitative or ethnograph* or fieldwork or "field work" or "key informant").tw.
121. interviews as topic/ or focus groups/ or narration/ or qualitative research/
122. (synthesis and qualitative studies).tw.
123. (synthesis and qualitative research).tw.
124. (systematic review and qualitative studies).tw.
125. (systematic review and qualitative research).tw.
126. critical interpretive synthesis.tw.
127. (meta and ethnography).tw.
128. meta-study.tw.
129. thematic synthesis.tw.
130. meta-narrative.tw.
131. textual narrative synthesis.tw.
132. framework synthesis.tw.
133. (grounded theory and synthesis).tw.
134. (triangulation and qualitative and synthesis).tw.
135. or/119-134
136. Epidemiologic studies/
137. Exp case control studies/
138. Exp cohort studies/
139. Case control.tw.
140. (cohort adj (study or studies)).tw.
141. Cohort analy$.tw.
142. (Follow up adj (study or studies)).tw.
143. (observational adj (study or studies)).tw.
144. Longitudinal.tw.
145. Retrospective.tw.
146. Cross sectional.tw.
147. Cross-sectional studies/
148. or/136-147
149. randomized controlled trial.pt.
150. controlled clinical trial.pt.
151. randomized.ab.
152. placebo.ab.
153. drug therapy.fs.
154. randomly.ab.
155. trial.ab.
156. groups.ab.
157. or/149-156
158. exp animals/ not humans.sh.
159. 157 not 158
160. Economics/
161. "costs and cost analysis"/
162. Cost allocation/
163. Cost-benefit analysis/
164. Cost control/
165. Cost savings/
166. Cost of illness/
167. Cost sharing/
168. "deductibles and coinsurance"/
169. Medical savings accounts/
170. Health care costs/
171. Direct service costs/
172. Drug costs/
173. Employer health costs/
174. Hospital costs/
175. Health expenditures/
176. Capital expenditures/
177. Value of life/
178. Exp economics, hospital/
179. Exp economics, medical/
180. Economics, nursing/
181. Economics, pharmaceutical/
182. Exp "fees and charges"/
183. Exp budgets/
184. (low adj cost).tw.
185. (high adj cost).tw.
186. (health?care adj cost$).tw.
187. (fiscal or funding or financial or finance).tw.
188. (cost adj estimate$).tw.
189. (cost adj variable).tw.
190. (unit adj cost$).tw
191. (economic$ or pharmacoeconomic$ or price$ or pricing).tw.
192. or/160-191
193. 5 and 24 and 59 and 82
194. 118 or 135 or 148 or 159 or 192
195. 193 and 194
